# Pituitary function following peptide receptor radionuclide therapy for neuroendocrine tumours

**DOI:** 10.1002/cam4.4345

**Published:** 2021-10-26

**Authors:** Marianne S. Elston, Amanda Love, Dev Kevat, Richard Carroll, Zhen Rong Siow, Sharon Pattison, Veronica Boyle, Eva Segelov, Andrew H. Strickland, David Wyld, Richard Gauci, Kim Kennedy, David Ransom, the Commonwealth Neuroendocrine Tumour Collaboration (CommNETs)

**Affiliations:** ^1^ Waikato Clinical Campus University of Auckland Hamilton New Zealand; ^2^ Department of Endocrinology and Diabetes Royal Brisbane and Women's Hospital Herston, Brisbane Queensland Australia; ^3^ Monash Health Victoria Australia; ^4^ Endocrinology Unit Capital & Coast Health Wellington New Zealand; ^5^ Department of Medicine, Dunedin School of Medicine University of Otago Dunedin New Zealand; ^6^ Monash University Clayton Victoria Australia; ^7^ Faculty of Medicine University of Queensland Brisbane Queensland Australia; ^8^ Fiona Stanley Hospital Perth Western Australia Australia

**Keywords:** hypogonadism, hypopituitarism, neuroendocrine tumours, radioisotopes, receptor, somatostatin

## Abstract

Peptide receptor radionuclide therapy (PRRT) is an increasingly used treatment for unresectable neuroendocrine tumours (NETs) that express somatostatin receptors. Normal pituitary tissue expresses somatostatin receptors so patients receiving PRRT may be at risk of developing hypopituitarism. The aim was to assess the prevalence of clinically significant hypopituitarism a minimum of 2 years following radioisotope therapy for metastatic NET. This was a multicentre study (Australia and New Zealand). Sixty‐six patients with unresectable NETs were included–34 had received PRRT and 32 comparison patients. Median follow‐up after PRRT was 68 months. Male hypogonadism was the most common hormonal abnormality (16 of 38 men [42%]) from the total cohort. Of these, seven men had primary hypogonadism (five from PRRT group) and nine had secondary hypogonadism (six in PRRT group). There was no difference in either male hypogonadism or other hormonal dysfunction between patients who had received PRRT and those that had not. Patients who have received PRRT out to 68 months following treatment do not show concerning hypopituitarism although there may be the suggestion of growth hormone deficiency developing. However, hypogonadism is common in men with NETs so the gonadal axis should be assessed in men with suggestive symptoms as the treatment of testosterone deficiency may improve the quality of life.

## Graphical abstract

Graphical representation of physiological pituitary activity on Gallium 68‐DOTA‐Octreotate PET/CT.

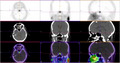

## 1 INTRODUCTION

Since the publication of the NETTER study in 2017, peptide receptor radionuclide therapy (PRRT) has become a standard of care for neuroendocrine tumours (NETs) patients who have progressed on a somatostatin analogue.^1^ PRRT is based on the principle that if a tumour expresses somatostatin receptors (SSTRs), particularly subtype 2a, a radiolabelled somatostatin analogue may be taken up by the tumour resulting in cell death. Some normal tissues also express SSTRs and show uptake on somatostatin receptor imaging, suggesting these tissues will receive a radiation dose from radiolabelled somatostatin analogue treatment. Normal adult human pituitary tissue expresses SSTR1, 2a and 5 and possibly SSTR3 and 4.^2^, ^3^, ^4^ Expression of the different receptor subtypes varies according to cell type within the pituitary with rat studies suggesting the highest SSTR2 expression in thyrotrophs and somatotrophs.^5^, ^6^ Whilst pituitary SSTR expression is well recognised by those involved in the care of NET patients, currently it is not known what dose of radiation is received by the pituitary during PRRT and whether this treatment could place patients at long‐term risk of hypopituitarism.

Hypopituitarism secondary to external beam radiotherapy has long been recognised and is important to identify as it may not only be associated with a decrease in quality of life but also premature mortality.^7^, ^8^ Effective treatment of pituitary hormone deficiency is readily available and considering the long survival of many patients with NETs, it would be important to identify if there is a correlative risk of hypopituitarism with PRRT. This will enable appropriate screening for and treatment of these deficiencies to improve the quality of life of patients. The onset of hypopituitarism may be insidious, therefore at‐risk patients require surveillance with serial testing. The risk of hypopituitarism increases with time following external beam radiotherapy with the extent and time of onset being dose‐dependent (reviewed in ref. [8, 9]). With lower dose external beam radiotherapy (<30 Gy) isolated growth hormone (GH) deficiency may be seen (the GH axis seeming to be the most radiosensitive), whereas high doses (>60 Gy) may result in a 30%–60% rate of gonadotropin, adrenocorticotropic hormone (ACTH) and thyroid‐stimulating hormone (TSH) deficiency after 10 years.^8^ However, age may influence the likelihood of specific axis dysfunction, with the GH axis potentially being more vulnerable in children than in adults.^8^, ^10^

To date, there have only been limited short‐term data (30 months post treatment) assessing pituitary function in patients who had received radioisotope therapy.^11^, ^12^, ^13^

The aim of this study was to assess the prevalence of clinically significant hypopituitarism in patients who were at minimum of 2 years following radioisotope therapy for metastatic NET compared to patients with metastatic NET who had not received PRRT.

## 2 MATERIALS AND METHODS

This was a multicentre (New Zealand and Australian centres) cohort study with eligible patients being those with unresectable NET who had either received PRRT a minimum of 2 years previously or had not received PRRT (comparison group). All patients who received PRRT had undergone prior functional imaging to confirm somatostatin‐avid disease (Krenning score >2). Patients were excluded if they had known primary pituitary pathology. Patients had an early morning (08:00–09:00 h) basal pituitary profile taken to measure: ACTH, cortisol, free thyroxine (FT_4_), TSH, follicle‐stimulating hormone (FSH), luteinising hormone (LH), estradiol (premenopausal women only), total testosterone (men only), sex hormone‐binding globulin, insulin‐like growth factor 1 (IGF‐1) and prolactin. Concomitant medications potentially influencing pituitary hormones were recorded (e.g. combined oral contraceptive pill, levothyroxine and somatostatin analogues) and the indication for therapy was noted (e.g. levothyroxine for primary hypothyroidism). Where applicable a menstrual history was obtained. All hormone results were reviewed independently by an endocrinologist followed by panel review by three endocrinologists (MSE, AL and RC) and categorised into normal or abnormal. In the event of an abnormal result, the test was repeated. Further investigations were performed and/or treated at the discretion of the treating endocrinologist according to a standard clinical care. All laboratory testing were conducted in facilities accredited for analysis of clinical samples (accredited by the national regulatory organisation National Association of Testing Authorities, and International Accreditation, Australia and New Zealand, respectively).

Hypopituitarism was defined as loss of one or more of the normal pituitary axes in the absence of confounding medical conditions/therapy. In the event of newly identified hypopituitarism, pituitary imaging was performed as per usual care to exclude any structural pituitary abnormality.

### 2.1 Ethics

Ethical approval was obtained from the relevant ethics committees (New Zealand [17/STH/23], Australia [HREC/17/QRBW/415; RES‐18‐756X]). Written informed consent was obtained from all patients.

### 2.2 Statistical analysis

Statistical analyses were performed using IBM SPSS Statistics for Windows, version 27.0 (IBM Corp. Released 2020: IBM Corp). Continuous data were tested for normality and non‐normally distributed data were analysed by the Kruskal–Wallis *H* test. Comparison of categorical variables, between groups, was analysed using the Fisher's exact test. A *p* value of <0.05 was used to reject the null hypothesis.

## 3 RESULTS

A total of 71 patients were recruited for the study. Of these, 38 patients had received PRRT and 33 served as the comparison group. Five patients were excluded from the study (four as they did not have pituitary function testing performed and one due to a pituitary mass diagnosed after baseline study investigations demonstrated an elevated prolactin and secondary hypogonadism, prompting a pituitary MRI). Patient demographic information is shown in Table 1. The PRRT group had a longer time from diagnosis (median 107 months vs. 24.6 months, *p *< 0.001) and were more likely to have received prior chemotherapy (*p *< 0.001) but otherwise were not significantly different from the comparison group. Of the 66 patients included in the study, 39 had normal pituitary function tests. Pituitary function results are shown in Table 2.

**TABLE 1:** Demographic information

	PRRT group, *N* = 34	Comparison group, *N* = 32	*p* value
Median age, years (IQR)	65.1 (56.1–71.7)	61.6 (54.9–68.7)	0.32
Male	23	15	0.14
Female (postmenopausal)	11 (9)	17 (13)	
Median BMI kg/m^2^ (IQR)	25.6 (23.3–30.6)	26.7 (21–31.2)	0.73
ECOG			
0	16	21	0.7
1	5	8	
2	2	1	
Not reported	11	2	
Somatostatin analogue use	18/34	20/32	0.47
Site of primary tumour			
Small bowel	13	17	0.4
Pancreas	18	10	
Lung	1	1	
Rectum	0	1	
Unknown	2	3	
Metastatic	33	32	0.3
Site of metastases			
Liver	29	23	0.2
Lymph nodes	11	20	0.01
Bone	6	4	0.6
Other	4	4	0.9
Carcinoid syndrome (small intestinal NETs)	8/13	7/17	0.05
Secretory syndrome (pancreatic NETs)	3/18	2/10	0.8
Median months since diagnosis, (IQR)	107 (67–132)	24.0 (1–56)	<0.001
Prior chemotherapy	17/34	1/32	<0.001
PRRT treatment cycles, median (IQR)	4 (4–4.25)	N/A	
1–2	2		
3–4	24		
5–6	7		
>7	1		
Median total dose, GBq (IQR)	31.8 (31.2–35.0)	N/A	
Median months from first cycle to bloods (IQR)	68 (51.3–102)	N/A	

Abbreviations: BMI, body mass index; ECOG, Eastern Oncology Cooperative Group performance status; IQR, interquartile range; N/A, not applicable; PRRT, peptide receptor radionuclide therapy.

**TABLE 2:** Pituitary function test results

Pituitary axis	PRRT group, *N* = 34	Comparison group, *N* = 32	*p* value
Cortisol (*n* = 65)^a^	1/33*	0/32	1.0
Thyroid (*n* = 58)^b^	1/28	1/30	1.0
Growth hormone	1/34^c^	0/32	1.0
Hypogonadal			
Male	11/23	5/15	0.51
Female	0/11	2/17	0.51
Prolactin (*n* = 65)^d^	4/34	2/31	0.67

^a^ One patient was excluded due to adrenal suppression from exogenous steroids; *cortisol normal on repeat testing.

^b^ Eight patients were excluded due to exogenous levothyroxine replacement for primary hypothyroidism started prior to NET treatment (six PRRT group and two comparison group).

^c^ One patient with confirmed growth hormone deficiency on glucagon stimulation testing.

^d^ One patient did not have prolactin analysed.

### 3.1 Adrenal axis

After exclusion of one patient who was receiving exogenous steroids, one patient from the PRRT group demonstrated a borderline low morning cortisol (140 nmol/L; RI 150–700 nmol/L) although this was normal on repeat testing (230 nmol/L). No difference was identified between the two groups (*p* = 1.0).

### 3.2 Thyroid axis

Eight patients were receiving exogenous thyroid hormone replacement for primary hypothyroidism before PRRT and so were excluded from analysis. Of these, six were in the PRRT group and two in the comparator group. Of the remainder, two patients had mildly abnormal thyroid function––one consistent with subclinical primary hypothyroidism not requiring treatment (PRRT group) and one patient in the comparator group showing a borderline low‐FT_4_ concentration with a TSH within the reference range.

### 3.3 Growth hormone axis

Initial assessment of the GH axis was performed by analysing IGF1. Overall, no differences in abnormal IGF1 concentration were identified between the two groups (*p* = 1.0). The median IGF1 *z*‐score was −0.3 in the PRRT group compared to 0.44 in the comparison group (*p *= 0.051). Three patients had abnormal IGF1 concentrations––two in the PRRT group of whom one had a mildly low IGF1, and the other had an elevated concentration. In the latter case, this had decreased on repeat testing and was associated with an undetectable GH concentration, including on glucose tolerance test and no clinical evidence of acromegaly and was thought not clinically relevant. The patient with a low IGF1 level (measured 55 months since PRRT, cumulative dose 33.7GBq) underwent glucagon stimulation testing confirming the presence of isolated GH deficiency. One patient in the comparator group had a mildly elevated IGF1 concentration which normalised on repeat testing.

### 3.4 Gonadal axis

Sixteen of the 38 men demonstrated abnormalities of the gonadal axis. No men were receiving exogenous testosterone replacement. Of the 11 hypogonadal men in the PRRT group, 5 had results consistent with primary hypogonadism. Of these, four had received prior chemotherapy and four had testosterone concentrations which warranted consideration of testosterone replacement. Six had tests consistent with secondary hypogonadism (of which four required consideration of testosterone replacement). Of the men with secondary hypogonadism the median time from PRRT was 96 months (with the earliest at 45.3 months) as compared to a median time from PRRT of 68 months for the men with normal gonadal function or primary hypogonadism (*p *= 0.6). There was no difference between the men with secondary hypogonadism and those with normal gonadal function in total radiation dose received (32.1GBq vs. 32.5GBq, respectively; *p* = 1.0). Five men in the comparison group had hypogonadism; two had primary hypogonadism and three had mild secondary hypogonadism, one of whom required consideration of testosterone replacement. The two patients with primary hypogonadism had not received chemotherapy. There were no differences in the proportion of patients with secondary hypogonadism between the PRRT and non‐PRRT groups (*p* = 1.0). The proportion of patients with hypogonadism was similar between the PRRT and non‐PRRT groups (*p* = 0.1) and PRRT was not a predictor of male hypogonadism (odds ratio 1.8 95% CI 0.5–7.1). In a logistic regression analysis including age, time from diagnosis, previous chemotherapy exposure and PRRT, no predictor was identified indicating a larger study is required to determine factors associated with hypogonadism in men with NET.

Two postmenopausal women in the comparison group had mildly low LH concentrations but no women had clinically relevant abnormalities of the gonadal axis. Median FSH concentrations were 61.5 U/L for postmenopausal women in the PRRT group versus 66 U/L for the comparator group.

### 3.5 Prolactin

Six patients had mildly elevated prolactin concentration, of whom four were in the PRRT group. There was no difference between groups (*p *= 0.67) and none were of clinical significance.

### 3.6 Posterior pituitary

No patients reported symptoms of diabetes insipidus to suggest posterior pituitary dysfunction.

## 4 DISCUSSION

The findings from this multicentre study of patients with a median follow‐up of 68 months following the first cycle of PRRT, are reassuring in that there was no increase in clinically relevant hypopituitarism when compared to a comparison group of patients who had not received PRRT.

Male hypogonadism was the most common hormone abnormality identified. However, this was seen in both groups and was a mix of both primary that is, testicular failure and secondary hypogonadism. Primary hypogonadism was identified in 7/38 men from the cohort and is not expected to be related to PRRT, illness or somatostatin analogues. Male hypogonadism (primary and secondary) is more likely in male cancer patients and cancer survivors, including those with NETs and treatment may improve body composition and potentially quality of life, although more data are still needed in this area.^14^, ^15^ Adequate testosterone concentrations are not only important for normal sexual function but also for energy, muscle strength and bone health.^16^ Importantly, testosterone deficiency is treatable with exogenous testosterone replacement. Men with suggestive symptoms including low desire, erectile dysfunction, fatigue, muscle weakness and/or suggestive biochemical findings such as unexplained normochromic normocytic anemia should be offered laboratory testing and consideration of treatment as clinically indicated.

Published research has suggested an increased risk of secondary hypogonadism in postmenopausal women who have received PRRT.^12^, ^13^ Teunissen and colleagues reported a reduction in gonadotrophin levels compared to baseline in a group of 21 postmenopausal women at 24 months post‐treatment with ^177^Lu‐octreotate.^12^ Similarly, Sundlov and colleagues also reported a non‐significant decrease in gonadotropin concentrations in postmenopausal women.^13^ Secondary hypogonadism in postmenopausal women is not clinically significant but is suggestive of a potential effect of PRRT on the gonadotrophs. Given the multicentre nature of the current study and the measurement of the female gonadotropins in several laboratories we were not able to confidently assess whether there was a difference in FSH and LH concentrations in postmenopausal women. However, no postmenopausal women in the PRRT group had low or inappropriately premenopausal values. Given the longer median time since treatment in the current study these findings are reassuring.

Sundlov et al identified a reduction in IGF1 levels over time since follow‐up, correlating with the number of treatment cycles and absorbed dose.^13^ However, it is well recognised that IGF1 decreases with age; so some of the changes seen may be normal age‐related change or due to illness rather than a direct radiation effect. Somatostatin analogues may also affect the somatotroph axis but in the Sundlov study the majority were already receiving these agents at baseline and the dose remained stable.^13^ We identified one patient with a clinically significant change in IGF1 levels in the PRRT group (with confirmed GH deficiency on glucagon stimulation testing), and on looking at patient *z*‐scores these showed a possible trend for being lower in the PRRT group. However, given that adult GH replacement therapy is contraindicated in patients with active malignancy,^17^ a finding of GH deficiency, whilst potentially explaining symptoms of excess fatigue and changes in body composition, would not alter the management for this group of patients. In the current study, we were primarily interested in clinically significant abnormalities with a potential for altering management and improving patient outcome such as quality of life rather than non‐clinically important, but statistically significant findings. The possible lower *z*‐scores in the PRRT group does support for the need for a further assessment of these patients, for example, after 10 years of follow‐up after PRRT to see if additionally, clinically significant, and potentially treatable pituitary dysfunction has developed over a longer timeframe.

The major limitation of this study is the lack of baseline pituitary function testing prior to PRRT. As such, a comparison group was included to try to account for the potential effects of illness from NET and ageing. In addition, laboratory testing was performed at the local hospital accredited laboratories rather than a single central laboratory. Subtle endocrine dysfunction may have been missed as routine stimulatory testing was not performed unless deemed clinically indicated by the treating endocrinologist. In particular, insulin tolerance testing, the gold standard for assessing cortisol and GH deficiency, carries a risk of serious harm, including death and is contraindicated due to age for most patients included in this study. Other groups looking at this area have also not routinely performed stimulatory testing of these patients.^11^, ^12^, ^13^ A strength of this study is the duration since PRRT with a median of 68 months (minimum 36 months) which is longer than that of previous studies in this area,^11^, ^12^, ^13^ even though the numbers we have included are still relatively low, demonstrating the difficulty in recruiting PRRT patients with a long duration of follow‐up. This is particularly important given that the external beam literature shows an increasing prevalence of pituitary hormone dysfunction over time, however a further study at 10 years should be considered in view of the external beam radiotherapy data.

## 5 CONCLUSION

Patients who have received PRRT out to 68 months following treatment do not show concerning hypopituitarism although there may be the suggestion of GH deficiency developing. Hypogonadism is common in men with NETs so the gonadal axis should be assessed in men with suggestive symptoms as treatment of testosterone deficiency may improve the quality of life.

## CONFLICT OF INTEREST

None to declare.

## ETHICS STATEMENT

Ethical approval was obtained from the relevant ethics committees (New Zealand [17/STH/23], Australia [HREC/17/QRBW/415; RES‐18‐756X]). Written informed consent was obtained from all patients.

## Data Availability

The data that support the findings of this study are available upon request from the corresponding author. The data are not publicly available due to privacy or ethical restrictions.
